# Case Report: Interstitial-intralesional laser therapy and laser-assisted new attachment procedure for the treatment of alveolar bone loss provoked by an aggressive pyogenic granuloma

**DOI:** 10.12688/f1000research.122693.2

**Published:** 2022-09-06

**Authors:** Anon Akkarapatum, Poramaporn Klanrit, Sajee Sattayut

**Affiliations:** 1 Oral and Maxillofacial Surgery, Faculty of Dentistry, Khon Kaen University, Khon Kaen, 40002, Thailand; 2 Lasers in Dentistry Research Group, Khon Kaen University, Khon Kaen, 40002, Thailand; 3 Oral Biomedical Science-Division of Oral Diagnosis, Khon Kaen University, Khon Kaen, 40002, Thailand

**Keywords:** pyogenic granuloma, interstitial-intralesional laser therapy, photocoagulation, LANAP, diode laser, tooth preservation

## Abstract

Background: A pyogenic granuloma (PG) is a common benign vascular lesion found in the oral cavity. The gold standard treatment of this lesion, comprising surgical excision and the elimination of etiological factors, cannot avoid tooth loss in the case of an aggressive pyogenic granuloma. Because of the prominent properties of 980 nm and 635 nm diode lasers in photocoagulation and photobiomodulation, we applied these wavelengths in the treatment of a large pyogenic granuloma with alveolar bone loss.

Case presentation: Our objective was to use a combination of interstitial-intralesional laser therapy, photocoagulation and laser-assisted new attachment procedure (LANAP) to preserve the teeth and periodontal tissue in a case of an aggressive pyogenic granuloma.

Results: The patient was a 13-year-old Thai male with a pyogenic granuloma involving the interdental papilla and lingual gingiva of the lower left first and second molars. The teeth were also displaced by the lesion. After treatment with three sessions of photocoagulation, three sessions of interstitial-intralesional laser therapy and two sessions of LANAP, the lesion was completely resolved. The periodontal status of the teeth was improved at the six-month follow-up.

Conclusion: The combination of interstitial-intralesional laser therapy, photocoagulation and LANAP was able to treat an aggressive pyogenic granuloma with tooth preservation.

## Introduction

A pyogenic granuloma is a lobulated exophytic lesion with a painless red erythematous papule. This lesion presents either as a pedunculated mass or with a sessile base. Pathogenic factors include chronic low-grade local irritation, hormonal factors and certain medications. ^1^ As this lesion is composed of a vascular component, blade excision leads to considerable bleeding and demands hemostatic intervention.

Near-infrared and red diode lasers provide favorable photocoagulation and photobiomodulation. These diode lasers are able to stimulate the formation of blood clots and promote healing after surgery. ^2^ Therefore, these wavelengths are widely used for the treatment of vascular lesions in the oral cavity via surface photocoagulation and interstitial-intralesional laser therapy. ^3^

Regarding periodontal disease treatment, laser-assisted new attachment procedure (LANAP) is able to stimulate the formation of new attachments. This technique also has advantages in hemostasis, granulation tissue removal and the reduction of periodontal disease pathogens. ^4^

Hence, we introduce an interstitial-intralesional laser technique for the treatment of aggressive pyogenic granulomas, aiming to treat lesions with minor gingival excision and preservation of periodontal tissue. Periodontal tissue recovery was achieved by LANAP. This case report presents a typical pyogenic granuloma with aggressive characteristics treated through the use of interstitial-intralesional laser therapy, photocoagulation and LANAP.

## Case report

**Figure 1.**
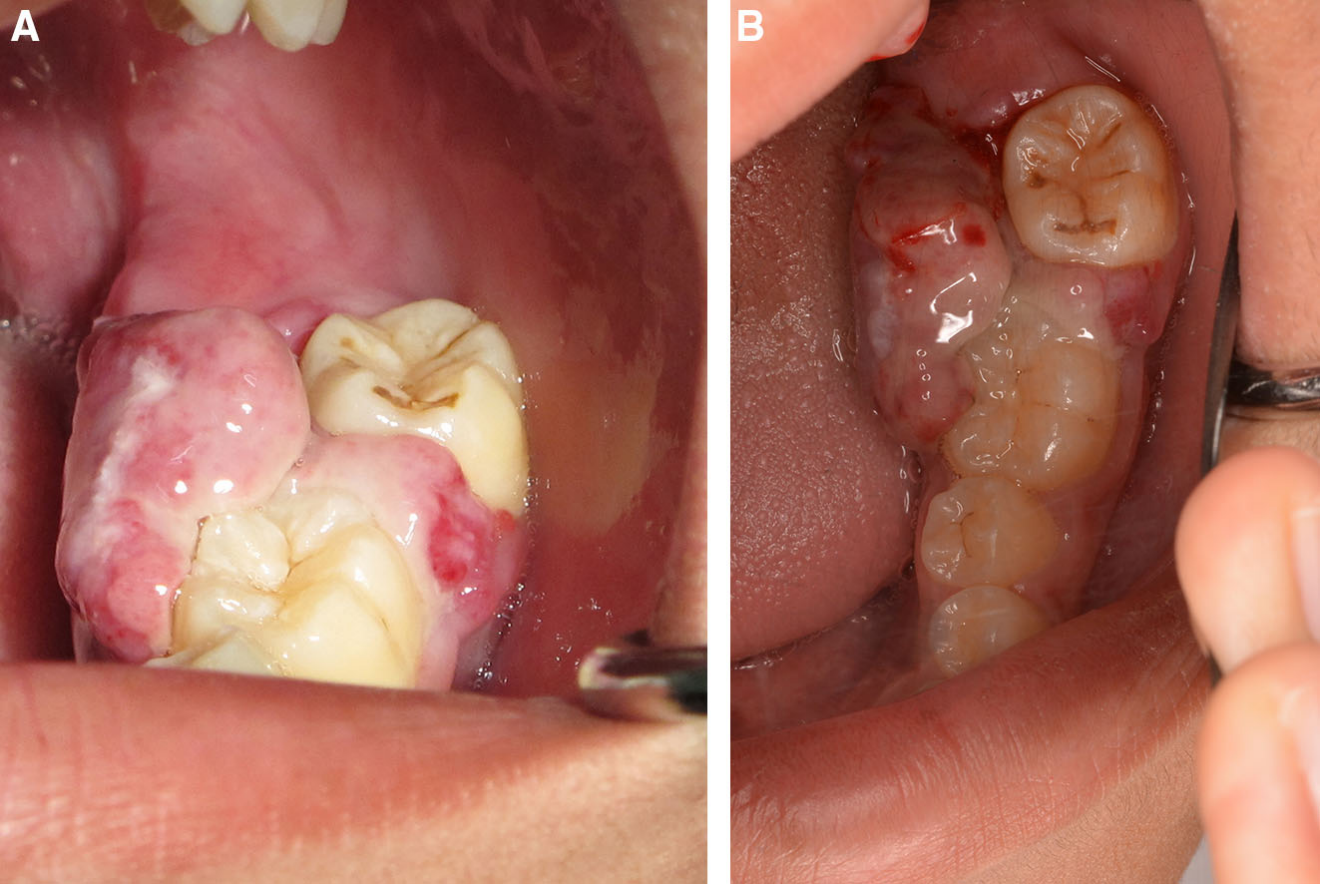
Clinical appearance. A. The erythematous pedunculated mass at tooth no. 36 and tooth no. 37. B. Tooth no. 37 was displaced from the normal position.

This case report was authorized by the ethics committee in human research, Khon Kaen University, reference number HE632040. The informed consent in Thai language from the patient and his parent was submitted to the ethics committee. The patient was a 13-year-old Thai male patient with a chief complaint of a rapidly swelling mass of the lower-left molar gingivae without pain for 10 days. There was no history of medical and psychological disorders of the patient and family. There was no systemic disease based on physical examination and laboratory investigation. The oral examination found an approximately 2×2 cm erythematous pedunculated mass with ulceration in the area of the interdental papilla and on the lingual gingiva of tooth no. 36 and tooth no. 37. The mass was soft consistency with no bleeding and no pus ( Figure 1A). Tooth no. 37 exhibited buccal displacement, as shown in Figure 1B. Tooth no. 36 and tooth no. 37 exhibited second-degree and third-degree mobility, respectively.

**Figure 2.**
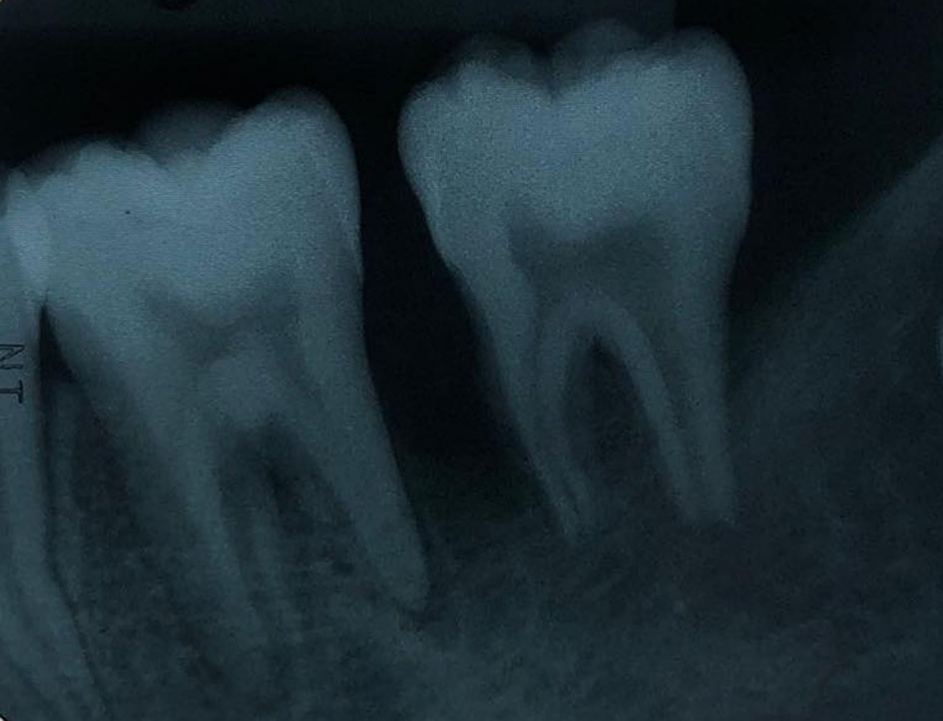
Radiographic examination. The periapical radiograph demonstrating alveolar bone loss at tooth no. 36 and tooth no. 37.

The periapical radiograph showed distinct periapical and proximal bone destruction of tooth no. 36 and tooth no. 37 ( Figure 2).

**Figure 3.**
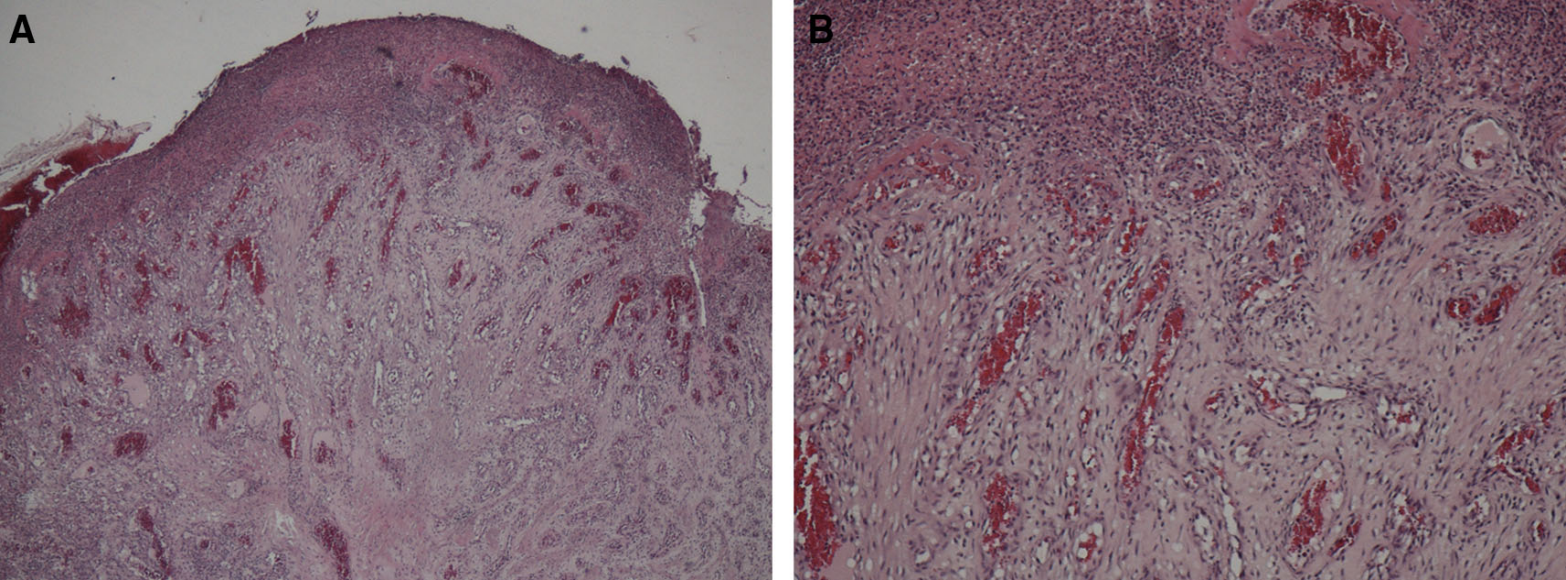
Pathohistological investigation. Histopathology section showing endothelial cell proliferation and distribution throughout the lesion, which are the characteristics of a pyogenic granuloma. A. At ×10 magnification and B. At ×40 magnification.

A sample obtained with an incisional biopsy using a 980 nm diode laser at 4 W continuous-wave with a 320-micron optical fiber confirmed the diagnosis of a pyogenic granuloma with histopathological features presenting endothelial cell proliferation, fibroblasts, neutrophils and chronic inflammatory cells in the connective tissue stroma ( Figure 3A and B).

**Figure 4.**
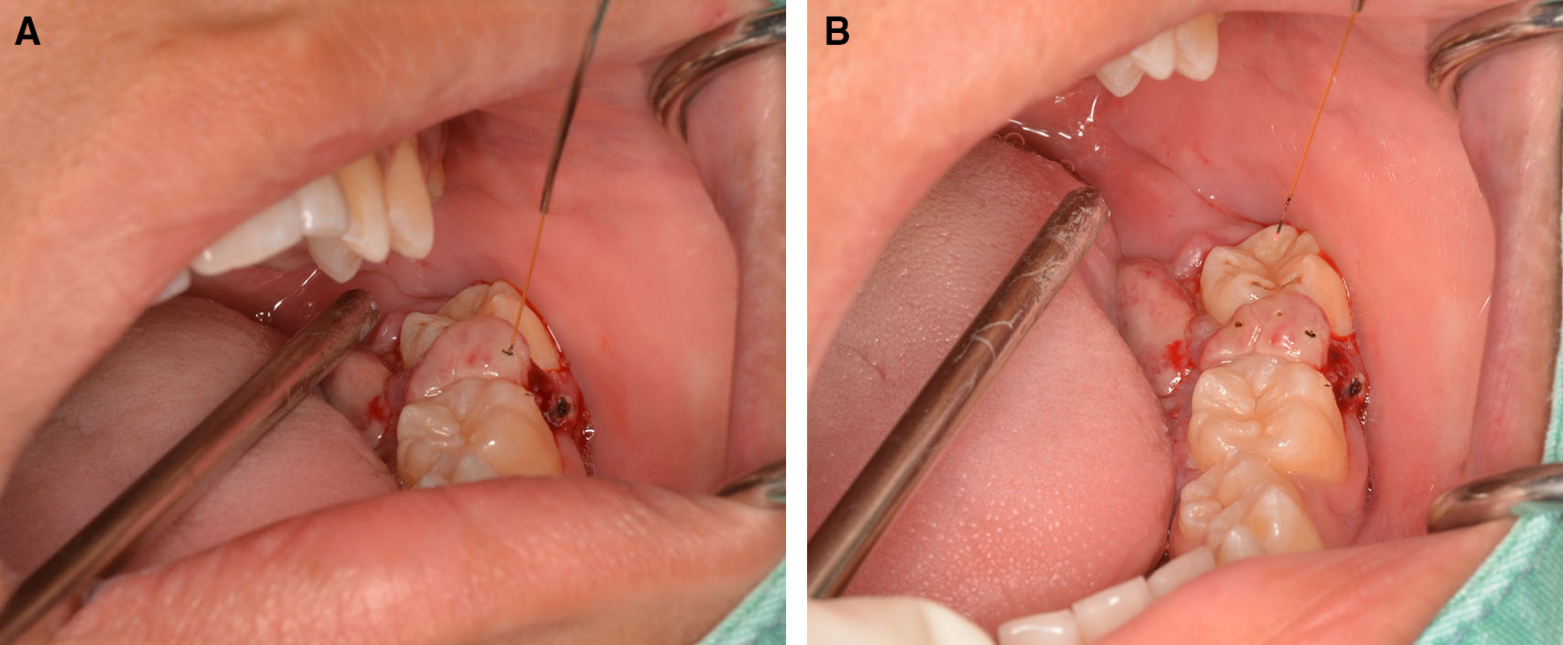
The interstitial-intralesional laser technique. A. The optical fiber was inserted into the lesion, beginning at the base of the lesion and then progressing to the top. The optical fiber was placed tangentially to the tooth and root surface. B. After a single treatment with interstitial-intralesional laser therapy on four areas.

The lesion was firstly treated by interstitial-intralesional laser therapy under local anesthesia using a 980 nm diode laser at 3 W continuous-wave with a 200-micron optical fiber, as shown in Figure 4A and B. After the insertion of the optical fiber tangentially to the tooth and root surface into the lesion, the laser was irradiated for five seconds. The lesion became pale and harder, indicating that coagulation was achieved.

**Figure 5.**
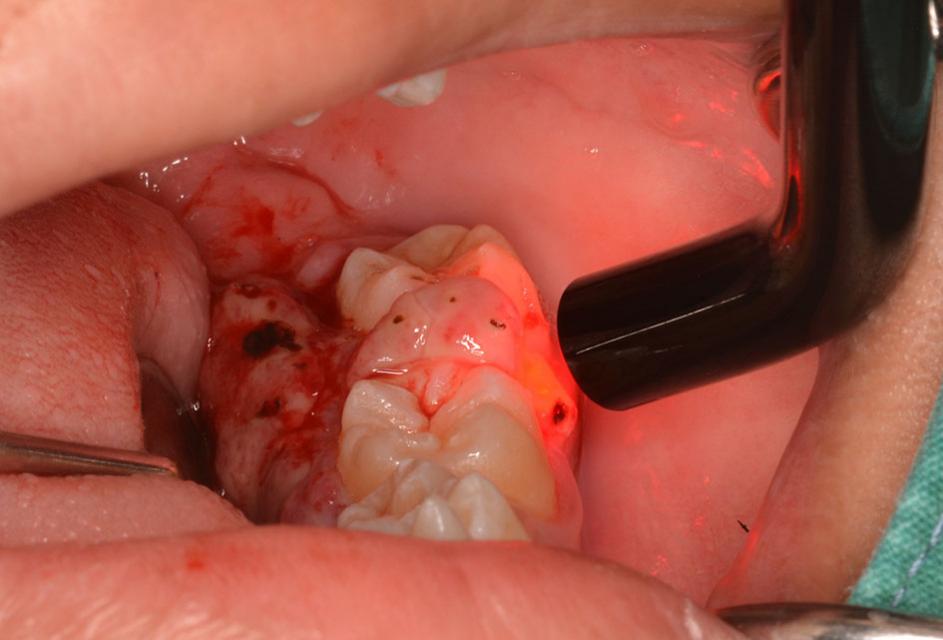
Photobiomodulation using a 635 nm diode laser via an 8 mm optical fiber.

This treatment was then immediately followed by treatment with using a 635 nm diode laser via an 8 mm optical fiber at 100 mW, continuous wave and 4 J/cm ^2^ with non contact mode to the lesion at the buccal and lingual sites for 1 session per area to achieve photobiomodulation and hemostasis effects ( Figure 5).

**Figure 6.**
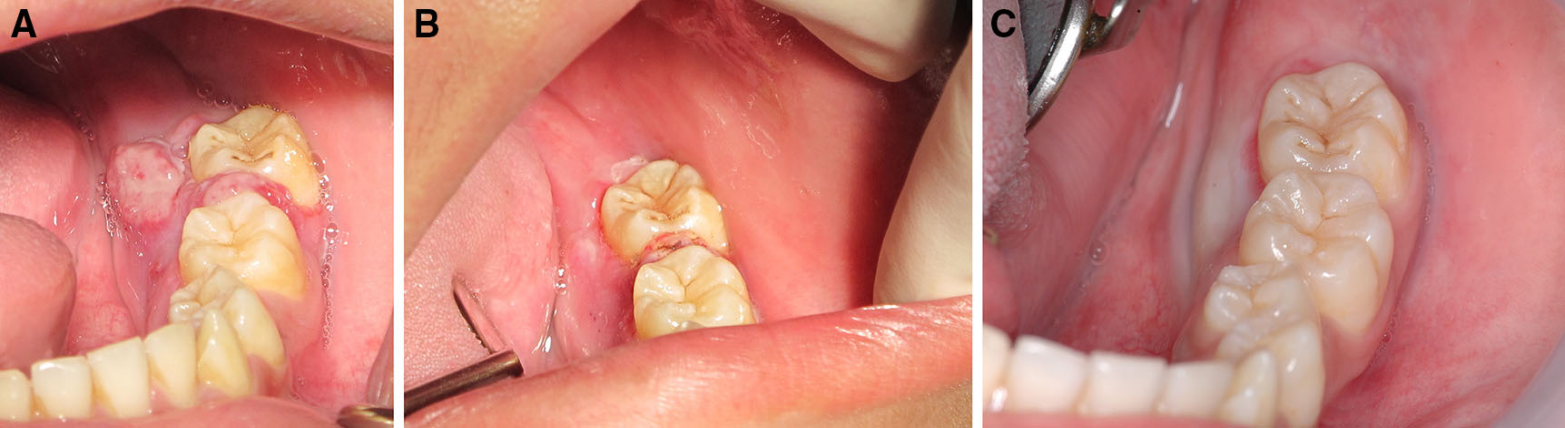
The clinical outcomes after interstitial-intralesional laser therapy. A. 2 weeks after the first therapy. B. 2 weeks after the second therapy. C. 3 weeks after the third therapy.

The patient was invited to have appointments for interstitial-intralesional laser therapy as previously described every two to three weeks. The remission of the pyogenic granuloma was observed, as shown in Figure 6A, B and C.

After two months of follow-up, the pyogenic granuloma involving soft tissue lesion was completely resolved. Tooth no. 37 returned to the normal position. The degrees of mobility of tooth no. 36 and tooth no. 37 were reduced to first-degree and second-degree mobility, respectively. The periodontal pocket was approximately 4 to 6 mm. The patient was treated with LANAP under local anesthesia to preserve the teeth.

**Figure 7.**
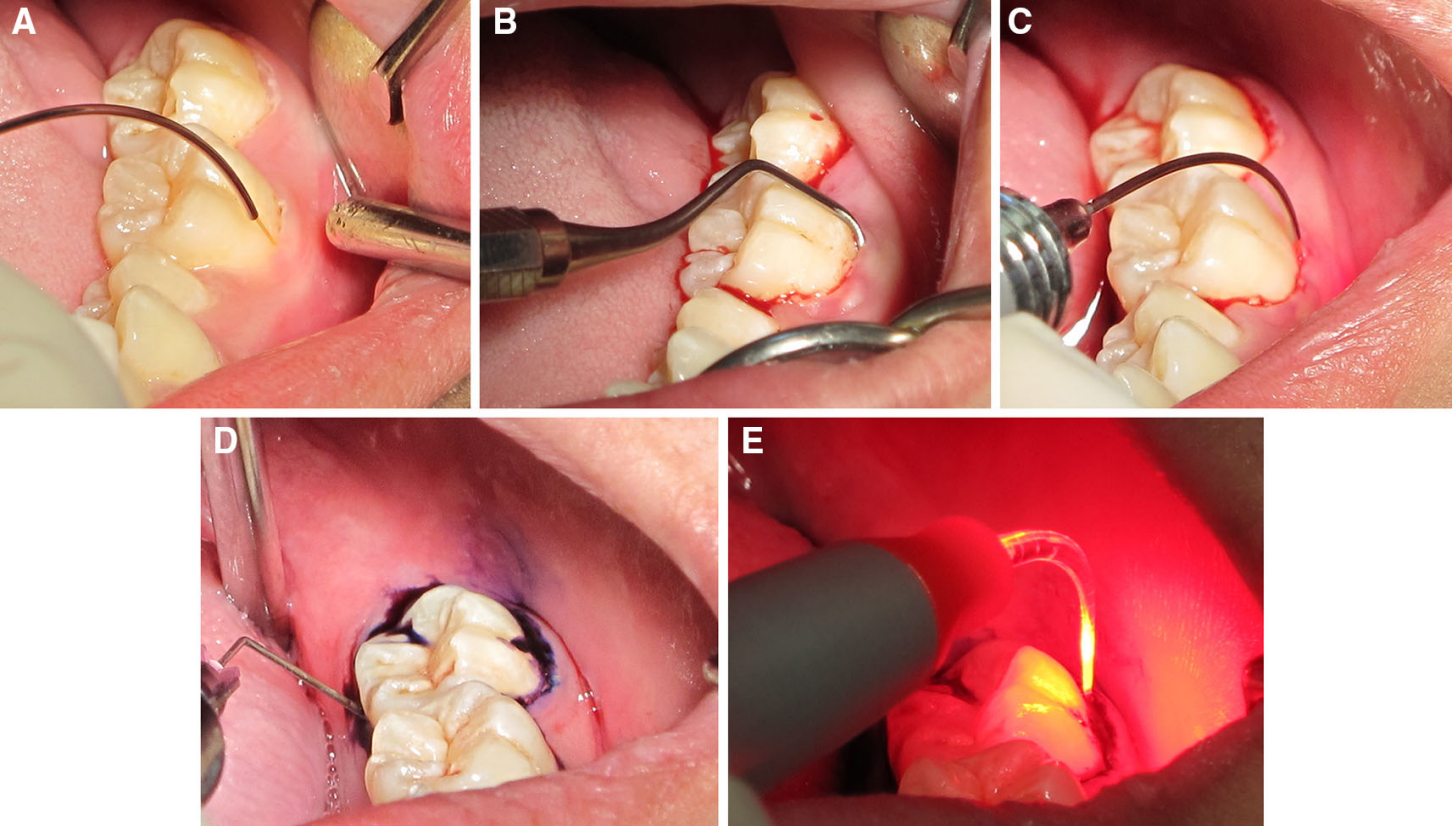
The LANAP procedure. A. The 980 nm diode laser was used for dilating the gingival sulcus. B. Scaling and root planning with hand instruments. C. The 980 nm diode laser was used for ablating the long junctional epithelium and granulation tissue in the gingival sulcus. D. Toluidine blue was injected as a photosensitizer into the gingival sulcus. E. The gingival sulcus was irradiated with a 635 nm diode laser via a 200-micron flexible tip to initiate photosensitizer.

The LANAP procedure consisted of three steps ^4^ as follows:

Step 1: After supragingival scaling with an ultrasonic scaler, a 980 nm diode laser at 0.7 W and continuous wave was delivered via a 200-micron optical fiber into the gingival sulcus ( Figure 7A). Then, it was followed by scaling and root planning ( Figure 7B).

Step 2: The epithelium and granulation tissue in the gingival sulcus was photoablated with a CW 980 nm diode laser at 2 W and continuous wave via a 200-micron optical fiber ( Figure 7C).

Step 3: Photodynamic therapy was administered using 0.1% toluidine blue as a photosensitizer and a 635 nm diode laser at 200 mW and CW for 15 sec via a 200-micron flexible optical fiber as a light source ( Figure 7D and E).

**Figure 8.**
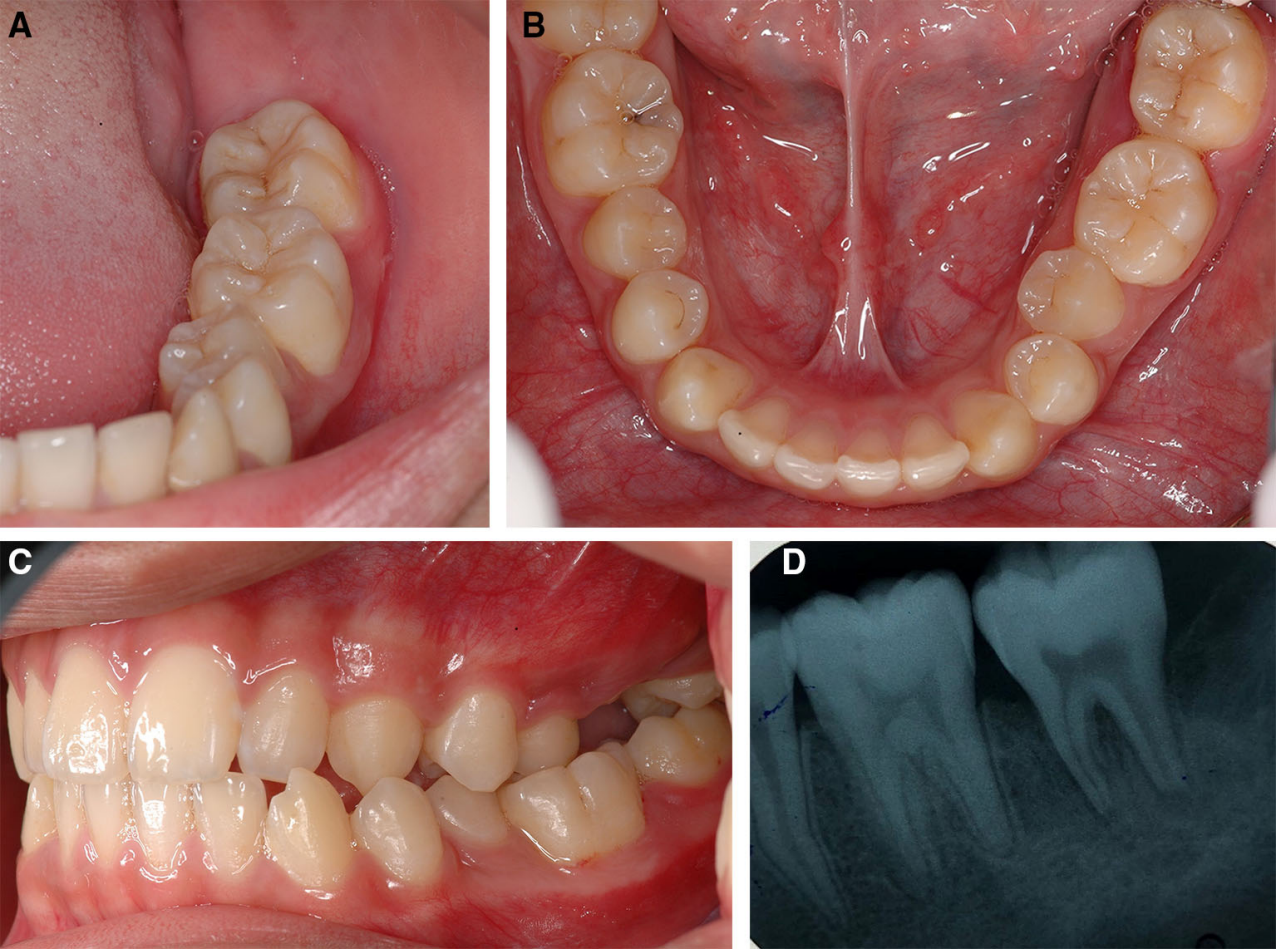
The clinical outcomes after LANAP procedure. A, B and C. Intraoral features after LANAP showing the normal appearance of the gingivae of the lower left molars and the repositioning of the teeth to their previous location and occlusion. D. Periapical radiograph of tooth no. 36 and tooth no. 37 showing an increase in periapical radiopaque characteristics in the previous defect.

After one month of LANAP, tooth no. 37 showed only first-degree mobility. No recurrence of the pyogenic granuloma was observed. The periodontal pocket depth was reduced, and no gingival recession of tooth no. 36 and tooth no. 37 was observed. The periapical radiograph showed improvement through the indication of bone formation at the periapical areas of tooth no. 36 and tooth no. 37, as shown in Figure 8. The second LANAP was conducted to maintain the periodontal status. There was no adverse and unanticipated event in overall treatments and outcomes.

The chronology of the treatment regime for this patient as follows:

1. interstitial-intralesional laser therapy with photobiomodulation
2. repeating treatments of interstitial-intralesional laser therapy for 2 sessions every two to three weeks
3. the LANAP at a two-month after the third session of the interstitial-intralesional laser therapy and
4. repeating the final LANAP a month after the first LANAP.

Due to the limitation of travelling from the COVID-19 pandemic, the patient was followed up by the dentist at his local health services. We followed the patient for another two sessions every three months for a- 6- month. There had been still no sign of recurrent of the lesion.

The patient and his parent were satisfied with the less invasion procedures and remission of the lesion with tooth preservation. Therefore, they allowed the authors as a team of surgeons to present and report this treatment for this may benefit the others who have the same condition.

## Discussion

The selection of laser wavelengths for biopsy and therapy is an important choice. An infrared diode laser was chosen in this case because of its low absorption by water of soft tissue and its capacity to generate more heat producing a deeper coagulative zone. This resulted in ablation with hemostasis. ^2^

In this case, using a 635 nm diode laser at a power less than 0.5 W not only initiated clot formation but also resulted in photobiomodulation, which allowed a positive response to the healing process, such as an increase in microcirculation, the stimulation of cell growth, and a reduction in inflammatory substances. ^5^

The treatment of the pyogenic granuloma in this report preserved the teeth and surrounding periodontal tissue. This outcome was different from that of a previous report in which the treatment of a pyogenic granuloma at tooth no. 11 in an 11-year-old female patient by total excision of the lesion resulted in gingival defects. The patient had to undergo free connective tissue graft. ^6^ Our technique with combined laser therapy showed no gingival defect after the resolution of the lesion.

Regarding the LANAP used in this case report, it was based on the techniques of De Angelis *et al.* ^4^ which combined photoablation and photodynamic therapy. This differed from other LANAP techniques by using only either Nd;YAG laser ^8^ or diode laser ^9^ for ablating epithelium and granulation tissue in the gingival pocket and root resurface.

In addition, there was a case report with a similar lesion: an aggressive pyogenic granuloma near the area of tooth no. 46 and no. tooth no. 47 in an 11-year-old female patient. With the use of surgical excision, tooth no. 46 near the lesion had to be extracted. ^7^ While in our patient, who was treated by a combination of interstitial-intralesional laser therapy, photocoagulation and LANAP, we were able to preserve the teeth and eliminate the lesion.

## Conclusions

From this report, a pyogenic granuloma with extensive periapical bone loss in a 13-year-old Thai male patient was treated with 980 nm and 635 nm interstitial-intralesional laser therapy, photocoagulation and LANAP. After six months of follow-up, there was no recurrence of the lesion and no complication of gingival recession. The periodontal status was improved. Therefore, we propose a combination of interstitial-intralesional laser therapy, photocoagulation and LANAP for the treatment of aggressive pyogenic granulomas to preserve the teeth involved in the lesions.

## Author contributions

AK: Data Curation, Investigation, Methodology, Visualization, Writing – Original Draft Preparation

PK: Data Curation, Investigation, Visualization, Writing – Original Draft Preparation

SS: Conceptualization, Methodology, Project Administration, Supervision, Writing – Review & Editing

## Data availability

All data underlying the results are available as part of the article and no additional source data are required.

## Reporting guidelines

Figshare. CARE flowchart. DOI: https://doi.org/10.6084/m9.figshare.20367705
