# Supplementary material for: Case Report: Interstitial-intralesional laser therapy and laser-assisted new attachment procedure for the treatment of alveolar bone loss provoked by an aggressive pyogenic granuloma
Source: F1000Res. 2022 Oct 7;11:883. Originally published 2022 Aug 2. [Version 3] doi: 10.12688/f1000research.122693.3 (PMC9453113; doi:10.12688/f1000research.122693.3)
Supplement: Peer review history

# Peer review: Reviewer response for version 3

**DOI:** 10.5256/f1000research.139459.r152706

**Published:** 2022-10-10

**Authors:** Samir Namour

**Affiliations:** 1 Laser Application in Dental Medicine, Department of Dental Sciences, Faculty of Medicine, University of Liege, Liege, Belgium

The manuscript can be indexed in this last version.

Are enough details provided of any physical examination and diagnostic tests, treatment given and outcomes?

Yes

Is the case presented with sufficient detail to be useful for other practitioners?

Yes

Is sufficient discussion included of the importance of the findings and their relevance to future understanding of disease processes, diagnosis or treatment?

Yes

Is the background of the case’s history and progression described in sufficient detail?

Yes

Reviewer Expertise:

Oral surgery,  laser dentistry

I confirm that I have read this submission and believe that I have an appropriate level of expertise to confirm that it is of an acceptable scientific standard.

# Peer review: Reviewer response for version 2

**DOI:** 10.5256/f1000research.138280.r149597

**Published:** 2022-09-07

**Authors:** Samir Namour

**Affiliations:** 1 Laser Application in Dental Medicine, Department of Dental Sciences, Faculty of Medicine, University of Liege, Liege, Belgium

Dear Editor,

 Authors perfectly adapted the new version of their manuscript.

 It still one correction to do before indexing:

 Erase the words " ***and hemostasis*** "  from the page 4, pargragrpah 5 ^th^:  The sentence becomes: ***“This treatment was then immediately followed by treatment with using a 635 nm diode laser via an 8 mm optical fiber at 100 mW, continuous wave and 4 J/cm2 with non contact mode to the lesion at the buccal and lingual sites for 1 session per area to achieve photobiomodulation effects (Figure 5).”***

 Because the energy of the Photobiomodulation is not high enough to produce homeostasis effect (very low energy are not able to generate heat). There is a bio-modulation effects only.

 There no need to reevaluate the manuscript again after this minor correction. The manuscript can be directly indexed.

Are enough details provided of any physical examination and diagnostic tests, treatment given and outcomes?

Yes

Is the case presented with sufficient detail to be useful for other practitioners?

Yes

Is sufficient discussion included of the importance of the findings and their relevance to future understanding of disease processes, diagnosis or treatment?

Yes

Is the background of the case’s history and progression described in sufficient detail?

Yes

Reviewer Expertise:

Oral surgery,  laser dentistry

I confirm that I have read this submission and believe that I have an appropriate level of expertise to confirm that it is of an acceptable scientific standard.

# Response

**Published:** 2022-10-04

**Authors:** Sajee Sattayut

**Affiliations:** Khon Kaen University, Thailand

We feel very grateful for your kind and keen suggestion. The modification was made as follows:-

 This treatment was then immediately followed by treatment with using a 635 nm diode laser via an 8 mm optical fiber at 100 mW, continuous wave and 4 J/cm ^2^ with non contact mode to the lesion at the buccal and lingual sites for 1 session per area to achieve photobiomodulation (Figure 5). It was interesting that an initial blood clot was also observed over the irradiated area.

 We just would like to report an interesting result on blood clot formation after photobiomodulation. Your suggestion is true. The dose which we used would not produce any photothermal effect.

# Peer review: Reviewer response for version 1

**DOI:** 10.5256/f1000research.134716.r146327

**Published:** 2022-08-22

**Authors:** Samir Namour

**Affiliations:** 1 Laser Application in Dental Medicine, Department of Dental Sciences, Faculty of Medicine, University of Liege, Liege, Belgium

Very interesting paper. Authors treated with success the problem of severe bone resorption and tooth mobility that can result in teeth loss by preserving the proximal part of the tumor to avoid food retention and pocket complication in case of total excision of the proximal part at the tumor at the first step of surgery.  This way offers a new and original way to treat this kind of risk. The paper is very interesting and an excellent and smart procedure to avoid future pocket complications and the loss of mobile teeth. Congratulations. To resume, this case report is bringing a new procedure and a new clinical management for the treatment of this kind of sensitive clinical cases. I highly recommend the indexing of this paper.

 However, we advise authors to do some adaptations to the manuscript:

1. Adaptation of the title to the content of the manuscript:

Actually, the title is : ***Case Report: Interstitial-intralesional laser therapy and laserassisted***

***new attachment procedure for the treatment of an aggressive pyogenic granuloma*. **My proposition is to make it as follow:  “ **Interstitial-intralesional laser therapy and laser-assisted new attachment procedure for the treatment of alveolar bone loss provoked by an aggressive pyogenic granuloma. Case Report”.**

1. In the case report description:
  - Second paragraph: the sentence is: “ *The periapical radiograph showed distinct periapical bone destruction of tooth no. 36 and tooth no. 37*” . We propose to add the words: and proximal in the sentence. The sentence becomes as follow: “ **The periapical radiograph showed distinct periapical and proximal bone destruction of tooth no. 36 and tooth no. 37** “
  - In the description of the photobiomodulation (PBM) paragraph:  Authors have to add, despite the description in the photo 5 showing the way of application, a descriptive text mentioning: sites of application of PBM  and the way of application, contact or non-contact, more details despite the photo. They have to also mention the total irradiation time per session, diameter of the fiber, etc.
  - In the same paragraph of PBM, authors have to erase the word “ **photocoagulation**”. Because the photobiomodulation can not deliver enough energy to heat tissue and to provoke any coagulate. It is a contradiction with the aim of the Photobiomodulation except if they omit to indicate the power used to induce a coagulation. The Diode (980 nm) can induce a coagulation under some irradiation conditions. The sentence becomes : “ *…continuous wave. and 4 J/cm2 to achieve photocoagulation and photobiomodulation effects (Figure 5)”.*
  - Authors have to add a paragraph with the Indication of the chronology of different treatments. Three procedures were used for the complete treatment: The interstitial-intralesional laser therapy, Photobiomodulation and LANAP were used in the treatment. Authors have to indicate clearly the chronology details. All of the 3 procedures were used in the same session or separately. We understand that Covid pandemic delayed some sessions, but authors should resume and indicate, in a separate paragraph the exact and advised chronology of 3 procedures per each session, the optimal timing for the different sessions and the chronology of the 3 procedures. Did they use the 3 procedures one after another in the same session or in separate sessions? What is the time between sessions? Their precise details and their news protocol has to allow other practitioners to perform similar procedures in order to obtain similar results.
2. In the discussion authors wrote :
  - First paragraph: “ *An infrared diode laser was chosen in this case because of its hemoglobin absorption ability. This resulted in ablation with hemostasis”. *We advise authors to adapt their sentence similar to this way: “ **An infrared diode laser was chosen in this case because of its low absorption ability  by water of soft tissue and its capacity to generate deeper and more heat. This resulted in ablation with hemostasis** *”.                          *
    In fact, the diode laser ( 980 nm) has low absorption by hemoglobin. But because of its low absorption by water and by consequence this low absorption by water allows this wavelength to have a deeper penetration in tissue resulting in an important heat generation allowing coagulation.
  - Second paragraph, the sentence: “ *For the photocoagulation technique, in this case, using a 635 nm diode laser at a power less than 0.5 W not only promoted clot formation but also resulted in photobiomodulation, …” *We advise authors to reformulate or to erase their paragraph because the Diode laser 635 nm (red light) at 0.1 W (energy delivered in the case report) cannot coagulate. This wavelength at 0.1 W can only induce a Photobiomodulation. To induce  a coagulation, the delivered energy has to be used at high output power. We advise authors to erase the word: “ **photocoagulate”** from their paragraph and replace it by “ **Photobiomodulation**”.

Again, congratulations to the authors for this new and very interesting clinical procedure. They are bringing to the lecturer a new way to treat this kind of disease avoiding the loss of teeth.

Are enough details provided of any physical examination and diagnostic tests, treatment given and outcomes?

Yes

Is the case presented with sufficient detail to be useful for other practitioners?

Yes

Is sufficient discussion included of the importance of the findings and their relevance to future understanding of disease processes, diagnosis or treatment?

Yes

Is the background of the case’s history and progression described in sufficient detail?

Yes

Reviewer Expertise:

Oral surgery,  laser dentistry

I confirm that I have read this submission and believe that I have an appropriate level of expertise to confirm that it is of an acceptable scientific standard, however I have significant reservations, as outlined above.

# Response

**Published:** 2022-08-28

**Authors:** Sajee Sattayut

**Affiliations:** Khon Kaen University, Thailand

We are very grateful to your kind and smart advices. Those provide this manuscript more meaningful. We will modify the manuscript as suggested and will back to reply to you.

# Response

**Published:** 2022-09-04

**Authors:** Sajee Sattayut

**Affiliations:** Khon Kaen University, Thailand

**Reviewer: Samir Nammour**

*Very interesting paper. Authors treated with success the problem of severe bone resorption and tooth mobility that can result in teeth loss by preserving the proximal part of the tumor to avoid food retention and pocket complication in case of total excision of the proximal part at the tumor at the first step of surgery.  This way offers a new and original way to treat this kind of risk. The paper is very interesting and an excellent and smart procedure to avoid future pocket complications and the loss of mobile teeth. Congratulations. To resume, this case report is bringing a new procedure and a new clinical management for the treatment of this kind of sensitive clinical cases. I highly recommend the indexing of this paper.*

*However, we advise authors to do some adaptations to the manuscript:*

*Adaptation of the title to the content of the manuscript:*

*Actually, the title is : Case Report: Interstitial-intralesional laser therapy and laser-assisted new attachment procedure for the treatment of an aggressive pyogenic granuloma. My proposition is to make it as follow:  “Interstitial-intralesional laser therapy and laser-assisted new attachment procedure for the treatment of alveolar bone loss provoked by an aggressive pyogenic granuloma. Case Report”.*

**Response: **The title was modified as your good suggestion.

*In the case report description:*

*Second paragraph: the sentence is: “The periapical radiograph showed distinct periapical bone destruction of tooth no. 36 and tooth no. 37” . We propose to add the words: and proximal in the sentence. The sentence becomes as follow: “The periapical radiograph showed distinct periapical and proximal bone destruction of tooth no. 36 and tooth no. 37“*

**Response:** More description of radiographic examination was wrote as you suggested.

*In the description of the photobiomodulation (PBM) paragraph:  Authors have to add, despite the description in the photo 5 showing the way of application, a descriptive text mentioning: sites of application of PBM  and the way of application, contact or non-contact, more details despite the photo. They have to also mention the total irradiation time per session, diameter of the fiber, etc.*

*In the same paragraph of PBM, authors have to erase the word “photocoagulation”. Because the photobiomodulation can not deliver enough energy to heat tissue and to provoke any coagulate. It is a contradiction with the aim of the Photobiomodulation except if they omit to indicate the power used to induce a coagulation. The Diode (980 nm) can induce a coagulation under some irradiation conditions. The sentence becomes : “ …continuous wave. and 4 J/cm2 to achieve photocoagulation and photobiomodulation effects (Figure 5)”.*

**Response:** More details of laser irradiation and application were added as indicated in red. It was noted that we applied this photobiomodulation to achieve hemostasis effect of the lesion.

 Therefore, the alteration was as below:-

 This treatment was then immediately followed by treatment with using a 635 nm diode laser via an 8 mm optical fiber at 100 mW, continuous wave and 4 J/cm ^2^ with non-contact mode to the lesion at the buccal and lingual sites for 1 session per area to achieve photocoagulation and photobiomodulation and hemostasis effects (Figure 5).

*Authors have to add a paragraph with the Indication of the chronology of different treatments. Three procedures were used for the complete treatment: The interstitial-intralesional laser therapy, Photobiomodulation and LANAP were used in the treatment. Authors have to indicate clearly the chronology details. All of the 3 procedures were used in the same session or separately. We understand that Covid pandemic delayed some sessions, but authors should resume and indicate, in a separate paragraph the exact and advised chronology of 3 procedures per each session, the optimal timing for the different sessions and the chronology of the 3 procedures. Did they use the 3 procedures one after another in the same session or in separate sessions? What is the time between sessions? Their precise details and their news protocol has to allow other practitioners to perform similar procedures in order to obtain similar results.*

**Response:** The treatment procedures were defined in chronologically in the part of the case report.

The chronology of the treatment regime for this patient as follows:-

1. interstitial-intralesional laser therapy with photobiomodulation
2. repeating treatments of interstitial-intralesional laser therapy for 2 sessions every two to three weeks
3. the LANAP at a two-month after the third session of the interstitial-intralesional laser therapy and
4. repeating the final LANAP a month after the first LANAP.

*First paragraph: “ An infrared diode laser was chosen in this case because of its hemoglobin absorption ability. This resulted in ablation with hemostasis”. We advise authors to adapt their sentence similar to this way: “ An infrared diode laser was chosen in this case because of its low absorption ability  by water of soft tissue and its capacity to generate deeper and more heat. This resulted in ablation with hemostasis”.*

*In fact, the diode laser ( 980 nm) has low absorption by hemoglobin. But because of its low absorption by water and by consequence this low absorption by water allows this wavelength to have a deeper penetration in tissue resulting in an important heat generation allowing coagulation.*

**Response:** We accepted your advice by altering the sentence as your suggestion.

 An infrared diode laser was chosen in this case because of its low absorption by water of soft tissue and its capacity to generate more heat producing a deeper coagulative zone. This resulted in ablation with hemostasis. ^2^

*Second paragraph, the sentence: “For the photocoagulation technique, in this case, using a 635 nm diode laser at a power less than 0.5 W not only promoted clot formation but also resulted in photobiomodulation, …” We advise authors to reformulate or to erase their paragraph because the Diode laser 635 nm (red light) at 0.1 W (energy delivered in the case report) cannot coagulate. This wavelength at 0.1 W can only induce a Photobiomodulation. To induce  a coagulation, the delivered energy has to be used at high output power. We advise authors to erase the word: “photocoagulate” from their paragraph and replace it by “Photobiomodulation”.*

**Response:** We do appreciate your advice to reserve photocoagulation to be a phenomenon from photothermal reaction only besides explaining the real clinical outcome of hemostasis effect. Therefore, we modified the sentences as below:

 In this case, using a 635 nm diode laser at a power less than 0.5 W not only initiated clot formation but also resulted in photobiomodulation, which allowed a positive response to the healing process, such as an increase in microcirculation, the stimulation of cell growth, and a reduction in inflammatory substances. ^5^

*Again, congratulations to the authors for this new and very interesting clinical procedure. They are bringing to the lecturer a new way to treat this kind of disease avoiding the loss of teeth.*

**Response:** We deeply appreciated your kind consideration and suggestion.

# Peer review: Reviewer response for version 1

**DOI:** 10.5256/f1000research.134716.r146326

**Published:** 2022-08-17

**Authors:** Kenneth Luk

**Affiliations:** 1 Faculty of Dentistry, The University of Hong Kong, Hong Kong, China

In this case report, the treatment protocol with wavelengths were well thought out. Laser parameters and techniques were well documented. Patient management was comprehensive.

 Pulsed Nd:YAG (1064nm) laser for LANAP  protocol was cleared by the FDA in 2004. ^1^ Yukna demonstrated LANAP  protocol with Nd:YAG laser can produce new cementum-mediated connective tissue attachment. ^2^ Recently,  LANAP seem to be broadened to include near-infra-red diode wavelengths. ^3^

 In this report, 980nm diode laser was used for LANAP protocol in step 1 and 2. Photodynamic therapy (PDT) with 635nm red laser was used in step 3. The initial LANAP protocol uses only Nd:YAG laser and no PDT was required. It would be more appropriate to describe the LANAP protocol in this case report as a modified LANAP protocol.

 Alternative lasers for this case:

 Like 980nm, 810nm, 940nm and 970nm are also well absorbed by haemoglobin and melanin. They can also be used for ablation, coagulation and photobiomodulation.

 Instead of PDT, photothermal therapy can be considered using Indocyanine green as a photosensitiser with 810nm diode laser.

Are enough details provided of any physical examination and diagnostic tests, treatment given and outcomes?

Yes

Is the case presented with sufficient detail to be useful for other practitioners?

Yes

Is sufficient discussion included of the importance of the findings and their relevance to future understanding of disease processes, diagnosis or treatment?

Yes

Is the background of the case’s history and progression described in sufficient detail?

Partly

Reviewer Expertise:

Lasers in Dentistry

I confirm that I have read this submission and believe that I have an appropriate level of expertise to confirm that it is of an acceptable scientific standard.

## References

[1] : The LANAP Protocol: laser-assisted new attachment procedure. *International Journal of Periodontics and Restorative Dentistry* .2007;87 18092452

[2] : Histologic evaluation of an Nd:YAG laser-assisted new attachment procedure in humans. *Int J Periodontics Restorative Dent* .2007;27(6) :577-87 18092452

[3] : Diode laser assisted management of endo-perio lesion in maxillary incisor using LANAP: A Case Report. *International Dentistry South Africa* .2010;38-43

# Response

**Published:** 2022-08-28

**Authors:** Sajee Sattayut

**Affiliations:** Khon Kaen University, Thailand

Many thanks for your comment on expanding the details of LANAP in terms of the development of techniques. We will add these to the manuscript.

# Response

**Published:** 2022-09-04

**Authors:** Sajee Sattayut

**Affiliations:** Khon Kaen University, Thailand

**Reviewer: Ken Luk**

*In this case report, the treatment protocol with wavelengths were well thought out. Laser parameters and techniques were well documented. Patient management was comprehensive.*

**Response:** Many thanks for your kind consideration.

*Pulsed Nd:YAG (1064nm) laser for LANAP  protocol was cleared by the FDA in 2004.1 Yukna demonstrated LANAP  protocol with Nd:YAG laser can produce new cementum-mediated connective tissue attachment.2 Recently,  LANAP seem to be broadened to include near-infra-red diode wavelengths.3*

*In this report, 980nm diode laser was used for LANAP protocol in step 1 and 2. Photodynamic therapy (PDT) with 635nm red laser was used in step 3. The initial LANAP protocol uses only Nd:YAG laser and no PDT was required. It would be more appropriate to describe the LANAP protocol in this case report as a modified LANAP protocol.*

*Alternative lasers for this case:*

*Like 980nm, 810nm, 940nm and 970nm are also well absorbed by haemoglobin and melanin. They can also be used for ablation, coagulation and photobiomodulation.*

*Instead of PDT, photothermal therapy can be considered using Indocyanine green as a photosensitiser with 810nm diode laser*.

**Response: **Your suggestion on emphasizing the modification of LANPP technique used for this case report was added in a discussion.

 Regarding the LANAP used in this case report, it was based on the techniques of De Angelis el al ^4 ^which combined photoablation and photodynamic therapy. This differed from other LANAP techniques by using only either Nd;YAG laser ^8^ or diode laser ^9^ for ablating epithelium and granulation tissue in the gingival pocket and root resurface.

 Owing to this being a case report, we would like to reserve other types of lasers or photosensitizers to be claimed that these would be applied as similar results have been proved in this study. 

*References*

*1. Gregg II R. H: The LANAP Protocol: laser-assisted new attachment procedure. International Journal of Periodontics and Restorative Dentistry. 2007. 87*

*2. Yukna RA, Carr RL, Evans GH: Histologic evaluation of an Nd:YAG laser-assisted new attachment procedure in humans.Int J Periodontics Restorative Dent. 2007; 27 (6): 577-87 PubMed Abstract*

*3. Shetty A, Ramachandra V. K, Shubhashini N. S, Anjali K, et al.: Diode laser assisted management of endo-perio lesion in maxillary incisor using LANAP: A Case Report. International Dentistry South Africa. 2010. 38-43*

**Response**: We could not find your suggested references in the Pubmed database. The references in the Pubmed database that supported the same ideas as you suggested were included.

 Jha A, Gupta V, Adinarayan R: LANAP, Periodontics and Beyond: A Review. J Lasers Med Sci. 2018;9(2):76-81.

 Chandra S, Shashikumar P: Diode Laser - A Novel Therapeutic Approach in the Treatment of Chronic Periodontitis in Type 2 Diabetes Mellitus Patients: A Prospective Randomized Controlled Clinical Trial. J Lasers Med Sci. 2019;10(1):56-63.
